# Risk of second cancer following radiotherapy for prostate cancer: a population-based analysis

**DOI:** 10.1186/s13014-016-0738-z

**Published:** 2017-01-03

**Authors:** Nina-Sophie Hegemann, Anne Schlesinger-Raab, Ute Ganswindt, Claudia Hörl, Stephanie E. Combs, Dieter Hölzel, Jürgen E. Gschwend, Christian Stief, Claus Belka, Jutta Engel

**Affiliations:** 1 Department of Radiation Oncology, Klinikum der Universität, Ludwig-Maximilians-University, LMU Munich, Marchioninistr 15, 81377 Munich, Germany; 2 Munich Cancer Registry (MCR) of the Munich Cancer Centre (MCC) at the Department of Medical Informatics, Biometry and Epidemiology (IBE), University of Munich, Marchioninistr 15, 81377 Munich, Germany; 3 Department of Radiotherapy, Klinikum Schwabing, Kölner Pl 1, 80804 Munich, Germany; 4 Department of Radiation Oncology, Klinikum Rechts der Isar, Technical University (TU), Ismaningerstr 22, 81675 Munich, Germany; 5 Institute of Innovative Radiotherapy (iRT), Helmholtz Centre, Munich, Germany; 6 Department of Urology, Klinikum Rechts der Isar, Technical University (TU), Ismaningerstr 22, 81675 Munich, Germany; 7 Department of Urology, Klinikum der Universität, Ludwig-Maximilians-University, LMU Munich, Marchioninistr 15, 81377 Munich, Germany

**Keywords:** Second cancer, Radiotherapy, Prostate cancer

## Abstract

**Background:**

To investigate the risk of second cancer and radiation induced second cancer following prostate cancer radiotherapy.

**Methods:**

We compared men with radiotherapy only with those treated with radical prostatectomy only and those with radiotherapy after radical prostatectomy. Cumulative incidences of second cancers were calculated. Cox analyses were performed to identify determinants influencing second cancer incidence.

**Results:**

Nineteen thousand five hundred thirty eight patients were analyzed. Age and median follow-up differed significantly with radiotherapy only patients having the highest median age (70.3 years) and radical prostatectomy only patients the longest median follow-up (10.2 years). Ten-year cumulative incidence of second cancer was 15.9%, 13.2% and 10.5% for patients with radiotherapy only, radiotherapy after radical prostatectomy and radical prostatectomy only (*p <*0.0001). Increasing age and belonging to the radiotherapy only group were associated with a higher risk of second cancer—no significant increase was seen in radiotherapy after radical prostatectomy patients. A significantly higher rate of smoking related malignancies, like lung, bladder and non-melanoma skin cancer, was seen in radiotherapy only patients.

**Conclusions:**

No clear increase in radiation induced second cancer was found in patients after radiotherapy for prostate cancer. Whereas the rate of second cancer was increased in radiotherapy only patients, no such increase was seen in patients with radiotherapy after radical prostatectomy. The increase of second cancer following radiotherapy only is highly likely to reflect advanced age and lifestyle habits and comorbidities.

## Background

Radiation induced second cancers (RISC) are rare but relevant late effects of radiation therapy (RT) [1, 2]. In principle most of the knowledge regarding RISC is derived from epidemiological data of atomic bomb survivors, nuclear accidents and database analyses [3]. Since there are no biological markers available allowing for a precise discrimination between RISC and non-radiation induced second cancer (SC), all assessments are based on epidemiological and/or statistical analysis. In principle, RISCs are defined as those cancers occurring inside or close to radiation exposed regions (field congruence) [4] and after a longer lag time (>10 years in some publications even >15 years) [5, 6]. However, in real world settings the determination of SC risks—and especially RISC risks—after therapeutic exposures is strongly limited by several factors: Heterogeneous patient cohorts, small sample sizes, complex influence of confounders and lacking data. Nevertheless, in case of Hodgkin’s lymphoma and breast cancer several trials document an increase in SC and RISC [7–9]. The risk of lung cancer after breast RT is considerably low in non-smokers (Odds ratio 1), however an increase (25-fold) was detected in smokers [10]. Thus RISC in this setting is triggered mainly by synergisms with a strong confounding factor.

In case of prostate cancer (PCa) patients being long-term cancer survivors [11] no clear picture emerges from all available data [12, 13]. Previous reports on the incidence of RISC using either Surveillance, Epidemiology, and End Results (SEER) or registry information provide conflicting results, whether after RT an increased risk of SC in PCa patients exists [14–25] or not [26–28]. With the help of the population-based Munich Cancer Registry (MCR) we attempted to gain further insight into SC risk after RT of PCa.

## Methods

### Data collection

**Fig. 1:**
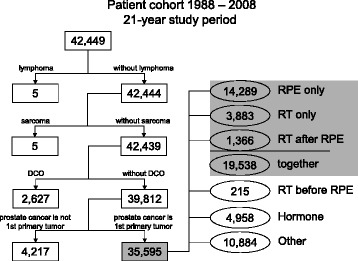
Patient cohort. DCO = death certificate only

The MCR is a population-based clinical cancer registry in Bavaria (Southern Germany) comprising 4.6 million inhabitants [29]. A total of 42,449 patients with PCa were registered from 1988 to 2008 (Fig. 1). Patients with lymphoma (*n =* 5) or sarcoma (*n =* 5) of the prostate, or with death certificate only (DCO) (*n =* 2,627) and men with evidence of a previous or synchronous malignant tumor (*n =* 4,217) were excluded. Of 35,595 patients with invasive PCa, 14,289 received radical prostatectomy (RPE only), 3,883 received radiation therapy (RT only) and 1,366 got radiation therapy after radical prostatectomy (RT after RPE). All analyses were performed on 19,538 patients (RPE only, RT only or RT after RPE). This analysis was in conformity with the Declaration of Helsinki (Sixth Revision, 2008) and was exempt from ethics approval. Bavarian state law allows the use of patient data for research, provided that person related data are made anonymous.

### Statistical analysis

The MCR organizes data in an Oracle database (Oracle, Belmont, CA) and uses Statistical Analysis Software, version 9.2 (SAS Institute, Cary, NC). The percentages of the presented subcategories are related to the sum of each item with available data; missing values are not taken into account. The significance level was set at 5%. Median follow-up was calculated for patients alive. To account for competing risks (e.g. death), the different age distribution between the three treatment groups and differences in follow-up cumulative incidence function according to Kalbfleisch and Prentice was used to calculate time to SC. Cancers occurring within the first year after treatment for PCa were not considered as SC. Differences in cumulative incidence among the three treatment groups were assessed by Gray’s test. Independent prognostic factors influencing time to SC were investigated by Cox proportional hazard regression. The proportional hazard assumption was visually checked by log-log plots. Hazard ratios, 95% confidence intervals (CI) and *p-*values acquired by Global Wald test are presented. The following factors were included simultaneously as independent variables modeling the incidence of SC in total in the multivariate analysis: age (<60, 60 – <65, 65 – <70, 70 – <75, ≥75 years), T- (T1, T2, T3, T4), N- (N0, N+, NX) and M-category (M0, M1) and treatment (RPE only, RT only and RT after RPE). Cox proportional hazard regression was also used to calculate models for the incidence of the most common tumor entities adjusted for the covariates mentioned above.

## Results

### Overall cumulative incidence and location of second cancer

**Fig. 2:**
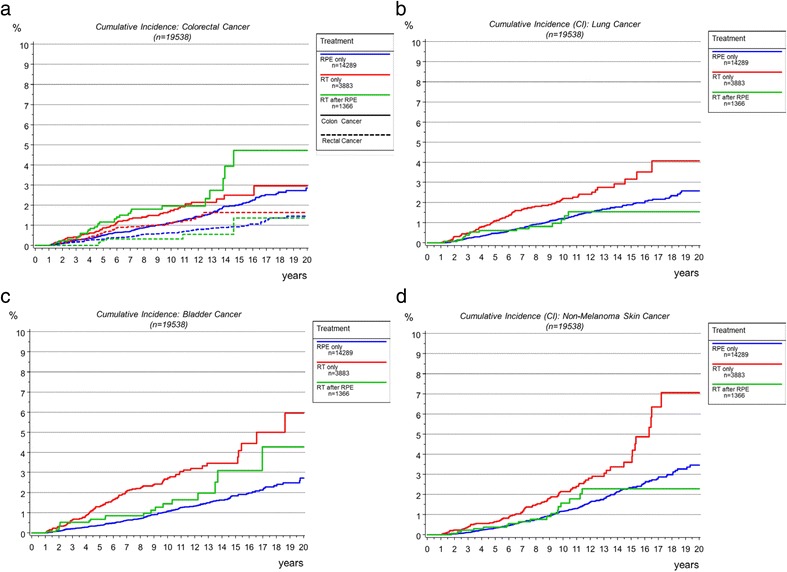
**a**-**d** Cumulative incidences of colorectal, lung, bladder and non-melanoma skin cancer stratified by treatment arms (RPE only, RT only, RT after RPE)

Table 1 lists the characteristics of the cohort with RT only or with RT after RPE compared to the surgical control group (RPE only). Patients with RT only had a higher median age (70.3 years) than patients with RPE only (65.2 years) or RT after RPE (64.4 years). Tumor characteristics were equally distributed between the three groups. Median follow-up between the three groups was significantly different with 10.2, 9.6 and 8.8 years in patients with RPE only, RT only and RT after RPE. Since previous studies on atomic bomb survivors estimated an average latency time of 5–15 years for RISC, analyses were done for SC occurring >10 years and >20 years [5, 6]. The cumulative incidence of SC was 15.9%, 13.2% and 10.5% after 10 and 26.7%, 26.6% and 23.7% after 20 years for patients with RT only, RT after RPE and RPE only (*p <*0.0001). The most frequent sites of SC were colon, rectal, lung, bladder and non-melanoma skin cancer (Table 2) significantly differently distributed between the treatment groups. After 10 years, colon cancer was observed with a cumulative incidence of 2.0% in RT after RPE followed by RT only (1.7%) and RPE only (1.1%) (Fig. 2a). Rectal cancer occurred more often in RT only (1.1%) than in RPE only (0.6%) and RT after RPE (0.3%) (Fig. 2a). The cumulative incidence of lung cancer was 2.2% after RT only and 1.2% in surgical patients regardless of postoperative RT (Fig. 2b). Bladder cancer was seen with a cumulative incidence of 2.7%, 1.5% and 1.1% in RT only, RT after RPE and RPE only (Fig. 2c). Non-melanoma skin cancer was observed with a cumulative incidence of 2.1%, 1.6% and 1.2% in RT only, RT after RPE and RPE only (Fig. 2d).

**Table 1:** Patient characteristics

Treatment	RPE only		RT only		RT after RPE	
	*N*	%	*N*	%	*N*	%
All	14289	100.0	3883	100.0	1366	100.0
Age [yrs]						
median	65.2		70.3		64.4	
< 60	3208	22.5	367	9.5	356	26.1
60–64	3758	26.3	599	15.4	364	26.6
65–69	4391	30.7	903	23.3	412	30.2
70–74	2432	17.0	1135	29.2	199	14.6
> =75	500	3.5	879	22.6	35	2.6
Risk Group						
low risk^a^	1843	13.2	529	17.0	35	2.6
intermediate risk^b^	3051	21.9	665	21.4	162	12.1
high risk^c^	6130	44.1	1067	34.3	504	37.8
locally advanced^d^	2615	18.8	738	23.8	522	39.1
advanced (N+)	275	2.0	108	3.5	112	8.4
metastasized (M1)	54	0.4	401	11.4	20	1.5
*Missing* ^*e*^	*321*	*2.2*	*375*	*9.7*	*11*	*0.8*
T-category						
T1	1852	13.4	586	19.2	146	10.9
T2	9152	66.0	1508	49.5	563	42.1
T3	2724	19.6	784	25.7	565	42.3
T4	139	1.0	170	5.6	62	4.6
*Missing* ^*e*^	*422*	*3.0*	*835*	*21.5*	*30*	*2.2*
N-category						
N0	9008	65.2	2046	67.3	869	64.6
N+	346	2.5	198	6.5	128	9.5
NX	4460	32.3	794	26.1	348	25.9
*Missing* ^*e*^	*475*	*3.3*	*845*	*21.8*	*21*	*1.5*
M-category						
M0	14235	99.6	3482	89.7	1346	98.5
M1	54	0.4	401	10.3	20	1.5
Second Cancer (*p <* 0.0001)						
developed	1876		638		186	
CI after 10 years		10.5		15.9		13.2
CI after 20 years		23.7		26.7		26.6
Median follow-up of survivors [yrs] (*p <* 0.0001)						
	10.2		9.6		8.8	

*CI* cumulative incidence

^a^low risk: PSA ≤ 10 ng/ml and Gleason score ≤ 6 and T1 to T2a (without T2)

^b^intermediate risk: PSA >10 – 20 ng/ml or Gleason = 7 or T2b

^c^high risk: PSA > 20 ng/ml or Gleason score ≥ 8 or T2c

^d^locally advanced: T3-4

^e^The percentage of the subcategories is related to the sum of each item with available data; missing values are not taken into account

**Table 2:** Cumulative incidence^a^ of most common cancer entities after 10 years of follow-up

	RPE only [%]	RT only [%]	RT after RPE [%]	*p-*value*
All second cancer	10.5	15.9	13.2	<.0001
Colon cancer	1.1	1.7	2.0	0.0017
Rectal cancer	0.6	1.1	0.3	0.0036
Lung cancer	1.2	2.2	1.2	<.0001
Bladder cancer	1.1	2.7	1.5	<.0001
Non-melanoma skin cancer	1.2	2.1	1.6	<.0001

^a^To account for competing risks, like death, cumulative incidence analysis was used to calculate time to second malignancy

*p-value was calculated by Gray-Test

### Factors influencing incidence of second cancer

Table 3 shows the results of the Cox analysis for SC in total with age, T-, N- and M-category and treatment as influencing factors. Age, T- and N-category and treatment were predictors for SC development. Increasing age was significantly associated with a higher risk of SC. RT only patients remained at a significantly higher risk of SC (HR1.404) compared to patients with RPE only (HR 1) or RT after RPE (HR 1.314). Already metastasized patients at initial treatment were at lower risk of SC due to their shortened life span. Analyzing the most frequent sites of SC separately adjusted for the abovementioned confounder variables treatment group remained a significant factor (Table 4). RT after RPE patients were at the highest risk of developing colon cancer compared to the other two treatment groups. Regarding rectal cancer, RT only patients (HR 1.751) were at highest risk and patients with RT after RPE were less at risk (HR 0.638) compared to RPE only patients (HR 1). RT only patients were at high risk (HR 1.733) of lung cancer during follow up. Hazard ratios regarding lung cancer of patients with RPE only (HR 1) or RT after RPE (HR 1.017) did not differ: Fig. 2b demonstrates no difference in cumulative probability of lung cancer after 10 years between patients with RPE only or RT after RPE. Likewise the risk of bladder cancer was significantly higher for RT only patients (HR 2.098) in contrast to a lower probability in patients with RPE only (HR 1) or RT after RPE (HR 1.601) (Fig. 2c). This was also observed for non-melanoma skin cancer (Fig. 2d) with RT only patients (HR 1.658) being at highest risk compared to the other two treatment arms (HR RPE only 1 and HR RT after RPE 1.305). Analysis of the most frequent sites of SC revealed a significantly increased rate of smoking and age related cancer entities, like lung, bladder and non-melanoma skin cancer in RT only patients.

**Table 3:** Cox proportional hazard model for second cancer with age, T-, N- and M-category and treatment as independent covariates

Patients: *n =* 19,538 Events: *n =* 2,700				
Covariates	HR	95%-CI		*p-*value*
Age [yrs]				<0.0001
< 60	1			
60–<65	1.278	1.124	1.453	
65–<70	1.618	1.434	1.826	
70–<75	2.136	1.881	2.425	
> =75	2.177	1.836	2.581	
T-category				0.0023
T1	1.089	0.964	1.231	
T2	1			
T3	1.204	1.095	1.324	
T4	1.086	0.786	1.501	
Missing	1.221	0.985	1.514	
N-category				0.0120
N0	1			
N+	1.095	0.863	1.388	
NX	0.965	0.885	1.052	
Missing	1.373	1.117	1.688	
M-category				0.6458
M0	1			
M1	0.913	0.619	1.346	
Treatment				<0.0001
RPE only	1			
RT only	1.404	1.268	1.553	
RT after RPE	1.314	1.127	1.531	

*HR* Hazard Ration, *CI* confidence interval

*p-value (Linear hypothesis test or Global Wald test)

**Table 4:** Cox proportional hazard model for selected second cancer entities adjusted for age, T-, N- and M-category and treatment as independent covariates

Patients: *n =* 19,538 Events: *n =* 2,700				
	HR	95%-CI		*p-*value*
Colon cancer				0.0035
RPE only	1			
RT only	1.326	0.970	1.813	
RT after RPE	1.903	1.269	2.853	
Rectal cancer				0.0077
RPE only	1			
RT only	1.751	1.179	2.601	
RT after RPE	0.638	0.278	1.465	
Lung cancer				0.0011
RPE only	1			
RT only	1.733	1.293	2.324	
RT after RPE	1.017	0.598	1.730	
Bladder cancer				
RPE only	1			<0.0001
RT only	2.098	1.585	2.778	
RT after RPE	1.601	1.001	2.561	
Non-melanoma skin cancer				
RPE only	1			0.0020
RT only	1.658	1.246	2.205	
RT after RPE	1.305	0.802	2.124	

*HR* Hazard Ration, *CI* confidence interval

*p-value (linear hypothesis test or Global Wald test)

## Discussion

The present study compares the incidence of SC in patients treated with RT only or RT after RPE to a surgical control group (RPE only). Two key findings emerged: First, the rate of smoking related cancer entities is increased in RT only patients but not in patients with RT after RPE. Secondly, patients who do receive RT as curative treatment are generally older probably harboring more comorbidities than patients undergoing surgery [30]. This implies that there are more confounders than age, T-, N- and M-category and treatment that influence the differing incidence of SC between the three treatment arms.

In accordance with literature [14–16, 18, 19, 24], an increased incidence of SC after 10 years has been seen in RT only patients (15.9%) vs. patients with RPE only (10.5%) or RT after RPE (13.2%). Differences in SC incidence between the cohorts should not be attributed to different median follow-up, as this has been taken into account by calculating cumulative incidence rates according to Kalbfleisch and Prentice. The cumulative incidence function by Kalbfleisch and Prentice does not only consider different follow up time but also considers the different age distribution between the three treatment groups and death as a competing risk factor. Cox analysis revealed that especially increasing age and being treated by RT only are the main predictive factors for a higher overall SC incidence.

When analyzing the situation for those SC sites in close proximity to the radiation portals (rectal, colon and bladder cancer) a complex picture emerges: At first glance, the rate of SC is increased after RT only – however no such increase is detectable in patients receiving both RPE and RT with the lowest absolute rate of rectal cancer being visible in patients having had both treatments. Based on pure reasoning, it seems therefore unlikely that radiation is really causal for the observed effect in RT only patients. The low number of events even in a large cohort will limit any valid risk assessment: In patients with RT only (HR 1.751) there was a 70% higher risk of rectal cancer compared to patients treated with RPE only (HR 1) and a less increased risk of colon cancer (HR 1.326 for RT only vs. HR 1 for RPE only). The diverging HRs of patients with RT after RPE with decreased risk of rectal (HR 0.638) and increased risk of colon cancer (HR 1.903) can be partially explained by the few events in the small group of patients with RT after RPE, which can also be seen in the wide 95% CI. Similarly, most other trials were not able to unequivocally document any increase in the incidence of colorectal cancer after RT [17, 25–27, 31–33]. Kendal et al. reported that an increased frequency of rectal cancer after irradiation is apparent on crude analysis, but age as an important confounder has to be taken into account. After adjustment for age these differences were no longer observable [26, 32]. Globally, our Cox regression analysis also revealed that age as a covariate generally plays a significant role in the development for SC at any site including colorectal SC (Hazard Ratio 1.278 in 60 - <65 year old patients vs. 2.177 in ≥75 year old patients, *p <*0.0001). Taken together our data do not indicate that RT is associated with a relevant risk of SC induction in the colorectum.

Similar to the colorectal mucosa variable parts of the bladder are exposed to radiation. Thus the bladder is also at risk for RISC. In our trial the 10-year cumulative incidence of bladder cancer was low with 2.7% in RT only, 1.5% in postoperative RT and 1.1% in RPE only patients. Cox analysis revealed that the RT only group (HR 2.098) had the highest risk of bladder cancer compared to the surgically treated patients. Similar to the findings for colorectal SC, combined treatment was associated with a lower incidence of bladder SC compared to RT only. Thus again our data failed to document an unequivocal increase in bladder RISC. In contrast the SEER analysis of Singh et al. showed a significant difference in the crude incidence rate of bladder cancer when comparing RT vs. surgery alone. On multivariate analysis in this study, age and irradiation were highly significant predictors of being diagnosed with bladder cancer [20]. As smoking data are absent from the SEER database, it was not possible to adjust for this confounder. In a recent report, Zelefsky et al. presented the 10-year likelihood rates for bladder cancer in patients treated with RPE or RT. One of the key messages of this trial is that patients treated with RT were older and had more serious comorbidities, many of them related to smoking. After Cox regression analysis, only age and history of smoking were significant predictors for the development of SC in this trial [27]. Likewise, Hamilton et al. came to a similar conclusion after comparing patients with brachytherapy and radical prostatectomy to the general population [34]: Older age and smoking were associated with an increased SC risk. Radical prostatectomy was not associated with a decreased pelvic malignancy risk, even when excluding patients with post-prostatectomy RT.

In addition to SC in close proximity to the primary radiation fields, several groups also explored the rate of SC outside the direct treatment areas.

In a recent report, Donin et al. presented the most common sites of SC among cancer survivors from SEER database [35]. Like in our cohort, the most commonly diagnosed SC among PCa survivors was lung (20.1%), colorectal- (15.5%) and bladder cancer (13.2%). Thus, smoking related cancers dominate the SC rates. A shortcoming of the data by Donin et al. is the missing correlation of SC rate with the respective treatment. The cumulative incidence of lung cancer in our cohort was 2.2% after RT only whereas it was the same (1.2%) in patients with RPE only or RT after RPE. Thus again, our data substantially indicate that SC in patients with RT only is related to a strong selection bias rather than indicating a real risk of SC after RT. Mainly patients being not fit for surgery will be selected for RT with smoking related disorders and age being major reasons [30, 36].

Smoking habits were not uniformly registered in MCR and could therefore not be used in the Cox analysis. We can therefore only hypothesize that smoking prevalence and its related comorbidities were higher in the generally older RT only patients. Depending on the prevalence, tobacco use alone could account for the observed 1.0% increase in cumulative incidence of lung cancer seen in the RT only group [37].

Apart from the abovementioned cancer entities, a single report documented an increase of non-melanoma skin cancer: Zelefsky et al. showed higher rates of skin cancer in patients with external beam RT compared to the general population or to patients who received brachytherapy. Apparently this is due to low-dose radiation from internally scattered X-rays, leakage of X-rays from the machine and/or neutron production, especially observed after doses ≥10 MV photons [38]. Apart from skin cancer Zelefsky et al. could not find any excessive risk of SC [21]. In contrast, no such increase was detectable in the RT after RPE group from our analysis indicating that the small increase in non-melanoma skin cancer in RT only patients may be attributed to other factors than the use of RT [39].

The high complexity of interacting confounding factors can be seen when the data of the large Prostate, Lung, Colorectal and Ovarian Cancer (PLCO) screening trial is taken into account: At first glance, patients after RT had increased risks for any SC (incidence 15.5/1000 person-years in irradiated patients vs. 11.4/1000 person-years in non-irradiated patients) with a substantial increase in lung cancer (RR 1.6, 95% CI 1.1-2.4). However, after adjusting for age, race, education, family history of cancer, COPD and smoking no increase for second colorectal or bladder cancer was documented any longer. Based on simple reasoning, it seems highly unlikely that no such increase occurs in close proximity to the radiation exposure, whereas a substantial increase occurs in far distant regions with only minimal radiation exposure. This interpretation is corroborated further by the fact that after breast irradiation with relevant part of the lungs being exposed, only a very low risk of SC is documented in non-smokers [10].

Most studies on RISC are based on pooled data sets. Inherently biases and confounders blur the interpretation of these results. A Dutch group analyzed the incidence rate of SC for patients prospectively treated in the TME trial, the PORTEC-1 and PORTEC-2 trial. The number of events (SCs) was much lower and however no significant increase for any pelvic or non-pelvic RISC was found [33].

All in all, up to now no consistent increased risk for SC after RT has been published. Most trials, like ours are based on retrospective data analyses with inherent shortcomings: Major confounders (e.g. smoking habits) have not been documented, treatment decisions (RT vs. no-RT) are mainly based on subjective and ill-defined parameters leading to strong biases, radiobiological inconsistences exist with patient receiving a combined treatment of RT and surgery displaying the lowest rate of SC in some data sets and crude SC rates outside the radiation fields are higher than in close proximity to the exposed areas. With RT only patients having the highest number of SC cumulative incidence, a correlation between dose and SC incidence in patients treated with RT only or with RT after RPE with assumingly different overall doses would have been interesting. Unfortunately in the MCR only limited information regarding the type of radiation delivery, the dosage and fields is given. We tried to overcome these shortcomings by using the cumulative incidence function according to Kalbfleisch and Prentice as an elaborate statistical method that accounts for death as a competing risk factor, the different age distribution and differences in follow-up between the three treatment arms.

In this regard a commentary from Kendal et al. on data provided by Moon et al. [40] nicely summarizes all these shortcomings with his own observation that no increase of RISC is detectable in 520,708 SEER cases with PCa after appropriate statistical considerations of age shifts (attained age) and other major factors [32]. Accordingly, Murray et al. [1] also pinpoints that differences between comparison groups, as well as differences in length of follow-up between treatment groups and failure to adequately correct for duration of follow-up may result in inaccurate conclusions.

## Conclusions

Our data suggest that RT does not increase the risk of SC. The increase of SC seen in patients with RT only in our cohort needs to be attributed to confounders suggested by an increased rate of smoking related malignancies. This interpretation is corroborated by the fact that no such increase was seen in patients treated with RT after RPE. At present we do recommend to inform patients about the negligible risk. However, it seems extremely important to ensure that the information given enables patients to realistically balance the value of the treatment against the risks. For any future trial it seems mandatory to carefully and prospectively document confounding comorbidities and lifestyle habits when attempting to gain a detailed and realistic insight into the risk of SC in PCA patients.

## Abbreviations

CI: Confidence intervals
COPD: Chronic obstructive pulmonary disease
DCO: Death certificate only
HR: Hazard Ratio
MCR: Munich Cancer Registry
PCa: Prostate cancer
PLCO trial: Prostate, Lung, Colorectal and Ovarian Cancer Screening Trial
PORTEC-1/2: Postoperative Radiation Therapy in Endometrial Carcinoma 1/2
RISC: Radiation induced second cancer
RPE only: Radical prostatectomy
RT: radiation therapy
RT after RPE: Radiation therapy after radical prostatectomy
RT only: Radiation therapy only
SC: Second cancer
SEER: Surveillance, Epidemiology, and End Results
TME: Total mesorectal excision
